# Stochasticity in microbiology: managing unpredictability to reach the Sustainable Development Goals

**DOI:** 10.1111/1751-7915.13575

**Published:** 2020-04-20

**Authors:** Jo De Vrieze, Thijs De Mulder, Silvio Matassa, Jizhong Zhou, Largus T. Angenent, Nico Boon, Willy Verstraete

**Affiliations:** ^1^ Center for Microbial Ecology and Technology (CMET) Faculty of Bioscience Engineering Ghent University Coupure Links 653 B‐9000 Gent Belgium; ^2^ Avecom NV Industrieweg 122P Wondelgem 9032 Belgium; ^3^ Department of Civil Architectural and Environmental Engineering University of Naples Federico II via Claudio 21 80125 Naples Italy; ^4^ Institute for Environmental Genomics Department of Microbiology and Plant Biology University of Oklahoma Norman OK 73019 USA; ^5^ Center for Applied Geosciences University of Tübingen Tübingen Germany

## Abstract

Pure (single) cultures of microorganisms and mixed microbial communities (microbiomes) have been important for centuries in providing renewable energy, clean water and food products to human society and will continue to play a crucial role to pursue the Sustainable Development Goals. To use microorganisms effectively, microbial engineered processes require adequate control. Microbial communities are shaped by manageable deterministic processes, but also by stochastic processes, which can promote unforeseeable variations and adaptations. Here, we highlight the impact of stochasticity in single culture and microbiome engineering. First, we discuss the concepts and mechanisms of stochasticity in relation to microbial ecology of single cultures and microbiomes. Second, we discuss the consequences of stochasticity in relation to process performance and human health, which are reflected in key disadvantages and important opportunities. Third, we propose a suitable decision tool to deal with stochasticity in which monitoring of stochasticity and setting the boundaries of stochasticity by regulators are central aspects. Stochasticity may give rise to some risks, such as the presence of pathogens in microbiomes. We argue here that by taking the necessary precautions and through clever monitoring and interpretation, these risks can be mitigated.

## Graphical abstract

In this review paper, we highlight the impact of stochasticity in single culture and microbiome engineering. Stochasticity may give rise to some risks, such as the presence of pathogens in microbiomes. We argue here that by taking the necessary precautions and through clever monitoring and interpretation, these risks can be mitigated.

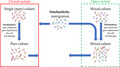

**Glossary**
Axenic: A closed ecosystem maintaining a single monoclonal population
Deterministic: The absence of randomness in the development of the microbial community structure and composition, which is mainly influenced by manipulatable niche‐based factors
Drift: The random fluctuation in the number of gene variations within a population over a certain period of time
Dynamics: The change in microbial community composition in function of time
Immigration: The addition of a specific taxon to a resident community from the species pool of the metacommunity
Invasion: The establishment of an immigrant taxon within a certain niche or ecosystem
Speciation: Genetic divergence within clonal populations as a result of lateral gene transfer and incorporation of foreign DNA through recombination, mutational divergence and natural selection
Stochastic: Random processes that influence the development of the microbial community structure and composition, which cannot be steered or controlled

## Introduction

For thousands of years, humans have been relying on microbial processes to produce a plethora of food products, such as bread, beer, vinegar, cheese and other fermented products. The industrialization of our society accelerated the development of novel technologies that directly exploit microorganisms and their unique abilities. For example, the treatment of wastewater by means of activated sludge and renewable energy recovery through anaerobic digestion is both established technologies, now in existence for over a century (McCarty, 1981; Sheik *et al.*, 2014). In our present society, drinking water and various foods contain microbial communities consisting of a variety of species that interact which each other. We define these cooperative assemblages of microorganisms as microbiomes (Burge, 1988). For future survival on this planet of our, at present 7.7 billion, but by 2050 predicted 9.7 billion co‐citizens (United Nations, 2019), we will depend on the production of these basic human goods through well‐designed and controllable systems (Kowalchuk *et al.*, 2008) in the framework of the Sustainable Development Goals. As long as we are unaware of the presence of a multitude of microorganisms in our surroundings and in what we eat and drink, we do not question the benefits of dealing with natural mixed and spontaneously evolving microbial systems or microbiomes.

Even though the discovery of microorganisms dates back to the 17th century with the design of a single‐lens microscope by Antonie van Leeuwenhoek (Lane, 2015), only in the last decades have we gained a view on the enormous microbial diversity in both natural and engineered ecosystems. The emergence of high‐throughput sequencing technologies has enabled us to characterize the microbial community composition, organization and dynamics, as reflected in the microbial resource management approach (Marzorati *et al.*, 2008), as well as identify potential key microorganisms in different systems. This has also allowed us to detect (potential) pathogenic species or genes in, for example, drinking water and microbial food products (Loman *et al.*, 2012; Marco *et al.*, 2017; Dowdell *et al.*, 2019). If we combine this knowledge with the fact that we are constantly in contact with microbiomes, for example through the consumption of fresh vegetables, and that such contact will increase due to the consumption of novel products of microbial fermentation, it becomes clear that societal and legislative concerns about the microbiome are imminent. This puts the use of microbiomes in engineered systems under pressure, despite their crucial role in our present society.

In the past decades, the fear for genetically modified organisms has had major consequences for the development of important applications of these powerful scientific tools. The issue of implementing microbiomes is entirely unrelated to the issue of GMOs, as no genes are modified, and all are 100% natural. Yet, one should be aware that the psychology of the consumer is sensitive to devious information. The purpose of this paper was to underscore the value of microbiomes that are not strictly defined nor strictly controllable in their evolvement. The analogy is that of a field of wheat: the farmer can never prevent that a poisonous plant species grows within his field. If regulators would impose such a request, normal agricultural practices would become impossible in terms of costs of preventing every single intruder and costs of controlling the absolute ‘purity’ of the crop (Chauhan *et al.*, 2017). The key feature is that one must deal with risks and this under conditions not entailing excessive costs.

Accurate control of microbial processes has been a central research topic for decades to improve process efficiency and/or accelerate production rates to maximize product quality and minimize operational costs. This ranges from basic grab‐sample pH measurements and subsequent base or acid addition to online sensorial analysis of multiple different parameters, for example in spontaneous fermented food products, to continuous steering of, for example, the oxygen supply in function of the incoming stream in activated sludge systems. However, there is a key gap between process stability, reflected in a constant output, and microbial community stability. A high degree of temporal variation has been observed in microbial community composition and organization, despite maintaining constant conditions, for example in anaerobic digestion (Fernandez *et al.*, 1999; De Vrieze *et al.*, 2013). A similar temporal variation was observed in full‐scale anaerobic digestion (De Vrieze *et al.*, 2016) and wastewater treatment systems (Meerburg *et al.*, 2016).

This temporal variation in microbial composition directly relates to the concept of deterministic vs. stochastic processes concerning microbial community organization. Deterministic or niche‐based factors strongly impact the way microbial communities are organized and functionally performing (Wang *et al.*, 2013; Zhou *et al.*, 2013b; Vanwonterghem *et al.*, 2014; Zhou *et al.*, 2014; Griffin and Wells, 2017; Zhou and Ning, 2017; Ning *et al.*, 2019), and these can be manipulated, to a major extent, by the process operators. In contrast, stochastic factors, such as genetic mutation, gene duplication, cell damage by radicals, die‐off, interspecies interactions, emigration, immigration and random drift also, have been shown to impact microbial community assembly and performance (Sloan *et al.*, 2006; Ofiteru *et al.*, 2010; Evans *et al.*, 2017) and are difficult to control. These stochastic factors are central to the neutral theory of biodiversity (Hubbell, 2001). At present, a consensus exists that both deterministic and stochastic factors shape microbial communities in natural and engineered systems (Van Der Gast *et al.*, 2008; Zhou *et al.*, 2013b; Xu *et al.*, 2019). Single (pure) or axenic cultures are also stochastic, since these may be subjected to various gene modifications, which cannot be predicted, as well as to various types of stress. Nonetheless, the level of unpredicted changes can be larger in systems open to the immigration of new species. Therefore, a key issue in engineered microbial processes, especially in food and drinking water niches, is monitoring and control of stochasticity to a high‐quality and safe end product.

The key objective of this study was to set a framework for the concept of stochasticity as an important process that shapes the microbiome in engineered systems. First, the concept of stochasticity in single (pure) cultures and microbiomes will be considered. Next, the mechanisms underlying stochasticity and the consequences (issues and opportunities) for engineered systems will be examined. Finally, the concept of stochasticity will be approached from a regulatory point of view for (in)direct human applications.

## The concept and mechanisms of stochasticity in microbial ecology

**Fig. 1:**
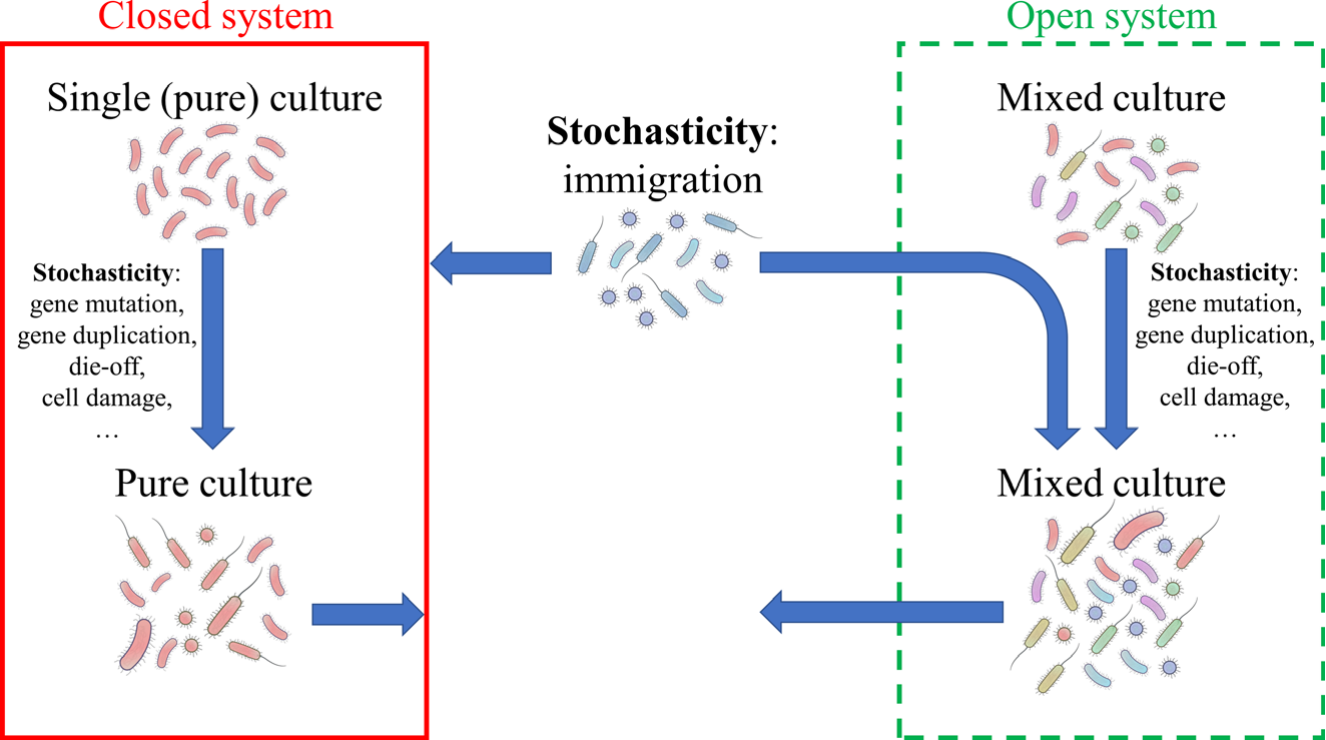
Schematic overview of the differential impact of stochasticity with respect to closed single culture and open mixed culture (microbiome) systems. The key difference is the contribution of immigration to open systems, in contrast to closed systems.

Fuelled by rapid developments in (meta)genomics, transcriptomics, proteomics and metabolomics techniques, greater attention has been paid to study microbial communities in their entirety and to identify the mechanisms that shape and influence microbial ecosystems of both single cultures and microbiomes. Mainly in the last decade, scientists started to acknowledge the importance of ecological stochasticity in shaping the microbial community (Zhou and Ning, 2017). Stochasticity differentially impacts closed single cultures and open microbiomes, given the absence of immigration in closed single culture systems (Fig. 1).

### Stochasticity at the cellular level: drift in single (pure) cultures

Several industrial processes, e.g., to produce amino acids, organic acids, antibiotics and enzymes (Jiang *et al.*, 2017) and microbiology research studies are carried out using axenic cultures, cultivated in a closed and homogenous environment. Such a single‐strain culture is not challenged by interspecies interactions and immigration and could, therefore, be presumed more stable over time, provided that the efforts to maintain axenic conditions prove successful. However, this does not eliminate stochasticity, as multiple other stochastic processes can influence the microbial community.

Nicholson (2019) stepped away from a deterministic view of the cell as a neatly organized machine, but instead emphasized the dynamic nature of its constitution, the fluidity of its components and the non‐linear stochasticity of its underlying processes. Stochasticity, or noise, in gene expression is the driving force behind phenotypic variation within the same strain of species (Elowitz *et al.*, 2002; Raj and van Oudenaarden, 2008), which seems an inherent survival strategy offering resilience and flexibility to a clonal population. Kussell and Leibler (2005) proposed that clonal bacterial populations can use stochastic phenotype‐switching as a response to random and infrequent environmental changes. The randomization of the phenotype, i.e., phenotypic plasticity (Kussell and Leibler, 2005; Fusco and Minelli, 2010; Ackermann, 2015), as a survival strategy is favoured compared to responsive adaptation when the random switching rate mimics the statistics of environmental changes (Kussell and Leibler, 2005). Many bacterial species can navigate environmental change by utilizing their diverse metabolic capacities (Meyer *et al.*, 2004; Swingley *et al.*, 2007; Narancic *et al.*, 2012), which provides these populations with a distinctive advantage over non‐versatile populations. It allows them to (i) persist at fluctuating conditions and (ii) divide labour between individual cells to increase the overall population function (Ackermann, 2015). It is well established that environmental factors can promote metabolic diversification, but also cell‐inherent dynamics can cause metabolic heterogeneity within clonal microbial populations (Takhaveev and Heinemann, 2018).

In contrast to phenotypic heterogeneity, genetic drift can promote genome reduction in a population in low fluctuating environments. New mutations (with a higher tendency for deletions) are more likely to become fixed when a bacterial lineage has to adapt to a novel lifestyle that reduces its long‐term population size, such as obligate symbiosis or a limited habitat range (Kuo *et al.*, 2009). For example, under constant ambient conditions (e.g. controlled reactor systems), bacteria with less coping mechanisms may outcompete and replace those that need to invest energy maintaining mechanisms that they do not require to survive under those specific circumstances. Thus, genetic drift can be a survival strategy when a single culture is subjected to fluctuating conditions.

### Stochasticity in natural mixed microbial communities or microbiomes

Nature is primarily stochastic. Flexibility is a key trait of microbial populations, consisting of a single species, as well as entire natural and engineered microbial ecosystems. In natural ecosystems, such as wetlands, soils, estuaries and marine environments, microorganisms occur in complex and multispecies microbiomes, which can vary greatly in composition, habitat, functionality and their relationships to the ambient environment. These communities can range from very few species to complex aggregates of trillions of cells and go through stages of community succession (Lyautey *et al.*, 2005; Datta *et al.*, 2016; Wright *et al.*, 2019). The complexity of community dynamics, even in low diverse communities, can be illustrated through the interactions of three common gut bacteria cultivated under well‐controlled *in vitro* conditions (D'Hoe *et al.*, 2018). The results showed the difficulty in predicting the winner without a predictive model, which should include both the internal metabolism of community members, as well as their response to interaction partners. Successes in modelling the behaviour of community dynamics (Song *et al.*, 2015; Song, 2018; Succurro and Ebenhöh, 2018) suggest that communities are shaped by deterministic and therefore predictive mechanisms. Such models (i) are limited by stringent biological assumptions (Succurro and Ebenhöh, 2018), (ii) group species in metabolic ‘guilds’ (Kettle *et al.*, 2015; Muñoz‐Tamayo *et al.*, 2016) or higher taxonomic levels (Cremer *et al.*, 2017) or (iii) only apply to select species within a composed (synthetic) community (D'Hoe *et al.*, 2018). This makes these models, despite their relevance to understand ecosystem functioning, often insufficient to accurately predict stochasticity in microbiomes.

The dynamics of any microbial community, reflected in successional shifts (both genetic and phenotypic), is powered by a broad array of deterministic factors, i.e., pairwise interactions that occur between species, such as (i) syntrophy (mutually dependent interaction) (Morris *et al.*, 2013; Schink and Stams, 2013), (ii) synergy (microbes supporting each other’s growth by creating favourable conditions) (Herschend *et al.*, 2018; Shaikh *et al.*, 2018), (iii) predation (Chen *et al.*, 2011; Fukami and Nakajima, 2011; Maslov and Sneppen, 2017) and (iv) competition for physical space and substrate (Hibbing *et al.*, 2010) and interactions that occur between microorganisms and their environment. Microorganisms can alter their environment through the production of various metabolites, the formation of flocs and biofilms, and precipitation or solubilization of reactive substances. Ambient conditions, such as pH, temperature and substrate availability, in turn, can influence the survival and growth rate of microorganisms. These multiple interactions and external influences, generally, do not destabilize microbial communities, because there is a constant pursuit for optimal functionality through a selection of species from a diverse pool of redundant microorganisms provided by the frequent influx of ‘immigrant species’ in an open system. This constant selection combined with a diverse microbial community allows that microbial assemblies, undergoing successions and evolving as complex communities, can tolerate a large level of stochasticity and still achieve constant high‐level performance. Several specific stochastic processes have been identified to govern microbial populations and induce structural fluctuations. Random immigration (Vuono *et al.*, 2016; Kirkegaard *et al.*, 2017; Mei and Liu, 2019) within open ecosystems is a key process that can alter community assembly through niche and fitness driven selection leading to invasion (Li *et al.*, 2019), combined with stochastic extinction (Kramer *et al.*, 2013), genetic drift and speciation (Hanage *et al.*, 2006). Limitation of dispersion can further increase stochasticity in community assembly and downplay the influence of environmental (deterministic) variables (Evans *et al.*, 2017).

It has been statistically concluded that stochastic processes shape the bacterial communities in the blood of pikas and their arthropod vectors (Li *et al.*, 2018), using the nearest taxon index (NTI) and mean nearest taxon distance (MNTD), as described by Stegen and colleagues (2013). The NTI values between −2 and 2 indicate stochastic community assembly, whereas NTI values less than −2 or higher than 2 indicate that deterministic processes play a more important role in structuring the community (Li *et al.*, 2018). On the other hand, Meyerhof and colleagues (2016) found that microbial communities in marine lakes were driven by the deterministic processes (all NTI > 4) of environmental selection, resulting from oxygen, salinity and pH gradients, which act as filters on microbial growth (Meyerhof *et al.*, 2016). Hence, the higher the harshness of environmental filters, the higher the dominance of deterministic processes (Chase, 2007; Meyerhof *et al.*, 2016). If a full physical barrier towards all other species is installed (i.e. in single culture systems), the level of determinism is at its maximum. However, even then, as indicated before, stochasticity cannot be fully excluded.

Microbiomes in industrial applications are governed by similar stochastic and deterministic mechanisms. Belgian Gueuze beers are traditionally produced by spontaneous fermentation, which is characterized by microbial succession over a 6‐month fermentation duration (Spitaels *et al.*, 2014). Many other food products, such as salami, sauerkraut, sourdough, surströmming and hákarl, wine, cheese, yoghurt and kefir, all rely on microbiomes for flavour, odour, texture and even shelf life. In vegetable fermentation facilities that produce spontaneously fermented sauerkraut, the raw vegetables and indoor environment and surfaces hosted distinct microbiomes, which were reflected in the final product (Einson *et al.*, 2018). In contrast, human contamination was found to have no effect on the final product. The general finding is that main bacterial species involved in such processes are re‐occurring, due to inoculum composition and fermentation conditions, resulting in highly similar community compositions within the same production process (Catzeddu *et al.*, 2006; Połka *et al.*, 2015; Shangpliang *et al.*, 2018). Nevertheless, diverse and dynamic communities are inherent to these fermentation processes and often contain a collection of low abundant (rare) and transient species (Połka *et al.*, 2015; Shangpliang *et al.*, 2018). Yet, the products itself are perceived as safe, and some even as health‐promoting (Marco *et al.*, 2017; Einson *et al.*, 2018), supported by a long history of trouble‐free consumption.

## The implications of stochasticity: benefits versus challenges

Stochastic behaviour can be modelled both at the population and ecosystem level (Ning *et al.*, 2019), yet the direction in which populations and ecosystems shift cannot be predicted. The inherent uncontrollability and limited predictability of stochasticity are both a strength and a weakness of microbial communities used in engineered systems. Engineered systems conventionally and intrinsically provide predictability and stability, and uncontrollable variation is undesirable. Yet, the fact that a system can evolve in a multitude of ways (i.e. demonstrating flexible behaviour) can be a strong asset to assure optimal process performance under changing conditions.

### The challenges of stochasticity

The limited degree of predictability of stochasticity can be considered at the genomic, phenotypic and ecosystems level and can lead to unforeseen deviations from the optimal process performance in an engineered ecosystem. This requires the need to take preventive actions to limit the effects of stochasticity and/or take necessary precautions to steer stochasticity towards a beneficial outcome.

#### Single cultures in the laboratory

At the genomic level, genetic drift as a consequence of multiple‐generation isolation leads to genetic impoverishment, which causes these so‐called ‘laboratory rats’ to lose at least part of their metabolic potential (Lenski and Travisano, 1994; Masel, 2011). This was demonstrated in a 10,000 generation experiment with 12 populations of *Escherichia coli*, in which a clear drift away from the ancestor was observed in the first generations (Lenski and Travisano, 1994). At the level of eukaryotes, 23 generations of *Chironomus riparius* resulted in a genetic impoverishment that could not be recovered by crossing different laboratory strains (Nowak *et al.*, 2007). Genetic drift in laboratory bacterial cultures could be prevented by (i) limiting the number of laboratory generations and/or (ii) keeping relatively large populations sizes (> 100) in the laboratory (Etzel and Legner, 1999).

#### Open systems are prone to stochasticity due to immigration and invasion

Open ecosystems that rely on microbiomes, such as activated sludge, anaerobic digestion, composting and the (human) digestive tract, imply that these are open to different stochastic processes. In that context, two features are of particular interest, which are (i) entry of outsiders in the microbial ecosystem and (ii) exit of insiders to the outside world. The potential downsides of such an open system are twofold.

**Fig. 2:**
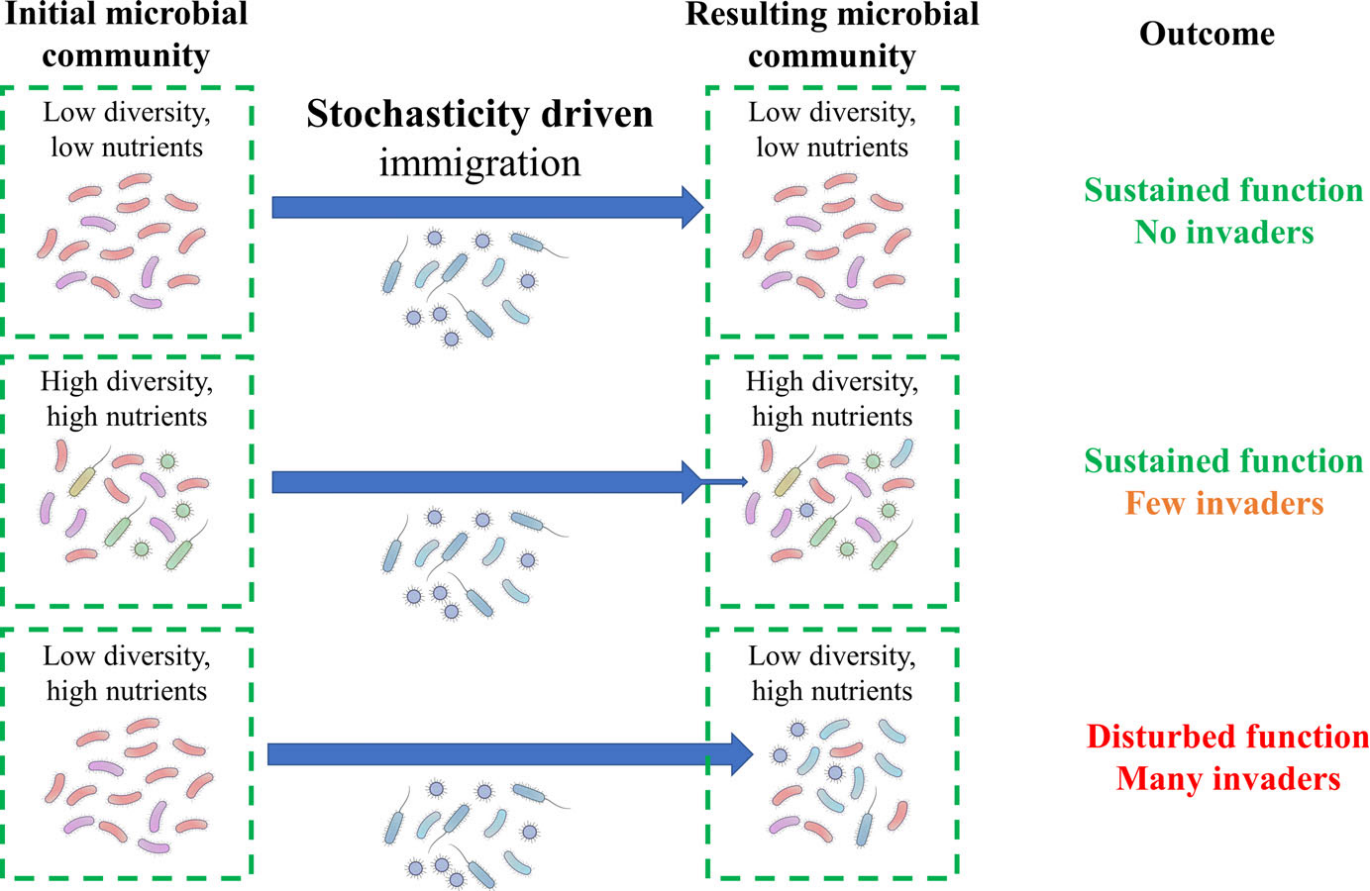
Visualization of the potential impacts of immigration in open systems in relation to nutrient availability and indigenous microbial community diversity. A low nutrient availability prevents the growth of invasive species, while a high indigenous microbial diversity promotes homeostasis to eliminate potential invaders.

Invasion of non‐desirable microorganisms could disturb process performance or compromise safety. Invasion can be defined as the establishment of an ‘immigrant’ species in a resident microbial community (Kinnunen *et al.*, 2016). This contrasts with immigration, which we define as the mere inflow, but not necessarily establishment, of species in a resident microbial community. Hence, invasion can be considered a consequence of immigration. Three key factors determine the potential degree of invasion. First, the presence or absence of environmental variables that induce stress in the microbial community strongly determines the susceptibility of the community to invasion. Under stress conditions, an invasive species could sustain process performance, while in the absence of such stress conditions, process performance can be impacted (De Roy *et al.*, 2013). Second, biodiversity in general (van Elsas *et al.*, 2012; Johnson *et al.*, 2013; Mallon *et al.*, 2015), and, more specifically, evenness (De Roy *et al.*, 2013), which also promotes process performance under stress (Wittebolle *et al.*, 2009), reduces the risk for and potential impact of invasion. Third, nutrient limitation also prevents the growth of potential invaders (Van Nevel *et al.*, 2013). Therefore, engineered ecosystems that are potentially susceptible to invasion‐induced process disturbance are those systems that have a low diversity with un‐occupied niches (i.e. tasks to be taken up) and a certain availability of nutrients. This implies that sterilized systems exposed to the open environment are extremely fragile, but also drinking water and composed culture‐based systems are prone to invasion and growth of outsiders (Fig. 2). An example is the natural birth of a ‘sterile’ human baby and the subsequent immediate colonization that follows until a mature human microbiome has developed (Koenig *et al.*, 2011). Another example is a well‐controlled system using composed cultures in the framework of the Micro‐Ecological Life Support System Alternative (MELiSSA) approach (Godia *et al.*, 2002). This approach (partially) relies on co‐cultures or single cultures in a bioregenerative life support system for complete recycling of gas, liquid and solid waste streams during space exploration (Hendrickx *et al.*, 2006). In such a system, heterotrophic strains need to be added to avoid/reduce invasion and subsequent process failure (Christiaens *et al.*, 2019). In contrast, even though immigration of species, which may lead to invasion, commonly takes place in open diverse systems, such as anaerobic digestion (Kirkegaard *et al.*, 2017) and activated sludge (Vuono *et al.*, 2016), mainly through the feedstock, this does not disturb and can even promote process performance.

In the case invasion does not disturb process performance, the introduction of new microbial species could have a direct or indirect impact to the outside environment in general and to the human health in particular. Invasion in systems in which not all niches are filled is reflected by the growth of non‐tuberculous mycobacteria (Dowdell *et al.*, 2019) and *Legionella* sp. (Lu *et al.*, 2016) in drinking water systems. The global occurrence of the protozoan pathogens *Cryptosporidium* and *Giardia* species in Western drinking water systems in the 1990s (Lechevallier *et al.*, 1991), and still commonly present in drinking water supplies in developing countries (Omarova *et al.*, 2018), indicates that such vulnerability to invasion remains a pressing issue. *Cryptosporidium* and *Giardia* species are also commonly present in ‘rich’ and diverse systems, such as wastewater sludge (Ramo *et al.*, 2017) and animal manure (Vermeulen *et al.*, 2017), and their removal in anaerobic digestion remains poorly documented (Nag *et al.*, 2019). A similar observation can be made for other pathogens, such as *Legionella* sp. (Viau and Peccia, 2009) and *Salmonella* sp. (Kjerstadius *et al.*, 2013) in digestate. Once present and capable to proliferate, such putative pathogens may reach the levels and physiological conditions that could impose a risk to human health, particularly if spreading could occur by aerosols (Brooks *et al.*, 2012).

Overall, in open systems, and even in well‐controlled semi‐closed systems, stochasticity, especially through invasion, effectively poses important challenges, but numbers need to be taken into consideration. Process failure is rarely an issue, but the level of putatively pathogenic species developing inside the system can be of concern in case of high infectivity, i.e., the probability of infection from exposure to one cell or particle (Rose and Gerba, 1991). By operating under conditions with limiting energy and nutrient sources, and/or conditions that sustain a high indigenous microbial diversity, stochasticity is not conflicting with reliability and safety, particularly concerning species that require high numbers to be infective.

### The benefits of stochasticity

Although stochasticity has downsides for single culture, composed culture and mixed culture (microbiome) engineered processes, the key advantages, especially towards microbiomes, are also twofold.

Homeostasis is the result of a dynamic balance of interactions between microorganisms in the community and with their environment (Li and Tian, 2016), which can be considered a key feature of invasion prevention. The constant threat of invasion keeps the system ‘alert’ towards hazardous invasions/disturbances that would impact process performance, due to the following two mechanisms. First, in a system that experiences a constant level of stochasticity, there is a higher probability that all niches are filled, thus, avoiding the intrusion of unwanted species (Ofiteru *et al.*, 2010). Second, stochasticity also maintains a certain level of process dynamics, even at highly stable and controlled operational conditions, thereby promoting the overall resistance of the process against disturbances (Briones and Raskin, 2003; De Vrieze *et al.*, 2013). Therefore, stochasticity can be considered a potential guardian of homeostasis, in addition to deterministic interactions between the microbiome and its environment.

Another key advantage of stochasticity resides in the fact that it sustains overall functional performance through the influx of novel species and/or creation of new niches, even though this strongly depends on the nature of the ecosystem (Marzorati *et al.*, 2008) and the spatial scale (Martiny *et al.*, 2011). It is generally assumed that a higher microbial diversity improves process stability and performance of engineered processes (Briones and Raskin, 2003; Beyter *et al.*, 2016) through a higher probability of positive species interactions, thus, positively affecting process performance (Cardinale *et al.*, 2002). Nonetheless, the benefit of diversity in microbial ecology is not a universal truth, as diversity assessment can be highly method‐dependent (Bent and Forney, 2008; De Vrieze *et al.*, 2018) and sampling‐related (Zhou *et al.*, 2011; Zhou *et al.*, 2013a). It only provides the onset of unravelling ecological mechanisms (Shade, 2017). A too high diversity could even increase antagonistic interactions (Becker *et al.*, 2012). The impact of stochasticity to functionality differs whether the microbial community is resistant, resilient or redundant in response to disturbances (Allison and Martiny, 2008). The key contribution of stochasticity to functionality resides in the increase of the redundancy potential of the microbial community. Community resistance (i.e. an unaltered microbial community in response to disturbances) appears to be independent of stochasticity. Community resilience (i.e. an altered microbial community that bounces back to its original composition) seems to be more depending on stochasticity though only if the temporal alteration sustains function. For community redundancy (i.e. a permanently altered community after a disturbance), stochasticity is key to create and sustain a plethora of novel community equilibrium compositions following a disturbance. Redundancy can be considered as key in microbiomes (Louca *et al.*, 2018). This is illustrated in anaerobic digestion (Langer *et al.*, 2015; De Vrieze *et al.*, 2017; Spirito *et al.*, 2018), where microbial communities continuously evolve as an inevitable consequence of stochasticity, but they nonetheless sustain functionality. Resilience and redundancy can both contribute to functionality within one microbiome. For example, syntrophic bacteria were found to be functional specialist and resilient, while higher level fermenters, such as Clostridia, were found to be functionally redundant within the microbiome of anaerobic granules for brewery wastewater treatment (Werner *et al.*, 2011).

Overall, stochasticity seems to be key to sustaining process performance by maintaining the microbial community ‘active’ (homeostasis) and ‘equipped to deal’ with to disturbances (redundancy). These examples show that stochasticity should be embraced for microbiome engineering as a natural mechanism of sustaining adequate process performance.

## How to deal with stochasticity: the regulators’ challenge

Stochasticity is part of life when dealing with both single cultures and microbiomes in biotechnological applications and process engineering. Thus, it requires a suitable framework for the regulatory authority to deal with stochasticity in an economically feasible and safe way. This is a twofold issue, as it requires (i) accurate monitoring of stochasticity and (ii) setting of boundaries on stochasticity, regarding the system in which it is considered. A proposal to solve both issues is formulated here.

### Monitoring stochasticity: a technological challenge

To monitor stochasticity, methods to detect changes in both single and mixed genetic and phenotypic traits are needed. Single culture phenotypic changes can be monitored through flow cytometry (Müller *et al.*, 2010) or matrix‐assisted laser desorption/ionization time‐of‐flight mass spectrometry (MALDI‐TOF MS) (Nowakiewicz *et al.*, 2017; Zhao *et al.*, 2017) as high‐throughput alternatives of conventional plating. Concerning the genetic level and the expression thereof, knowledge of the complete genome and plasmid genetic code is essential to avoid the potential expression of genes related to, for example, the production of toxins or antibiotic resistance in advance.

In contrast to single cultures that enable accurate monitoring of stochasticity, amongst others due to the elimination of immigration, this is much more challenging in microbiomes in which invasion is a key stochastic process. In an ideal situation, pathogenic microorganisms with their related virulence traits or antibiotic resistance genes (ARGs) should be absent in microbiomes in biotechnological applications. However, given the open feature of these systems, the invasion of potentially pathogenic species cannot be avoided and requires accurate monitoring.

A first monitoring approach concerns the ‘fingerprinting’ approach in which the degree of microbial community dynamics, i.e., the change in microbial community composition in function of time can be evaluated. Such a fingerprint and its evolution in function of time can be obtained on the genetic level through 16S rRNA gene amplicon sequencing (Pilloni *et al.*, 2012) or even simpler/older techniques, such as terminal restriction fragment length polymorphism (T‐RFLP) (Pilloni *et al.*, 2012; Camarinha‐Silva *et al.*, 2014; De Vrieze *et al.*, 2018), automated ribosomal intergenic spacer analysis (ARISA) (Gobet *et al.*, 2014; van Dorst *et al.*, 2014) or denaturing gradient gel electrophoresis (DGGE) (Marzorati *et al.*, 2008). Fingerprinting can also be carried out at the phenotypic level through flow cytometry (De Roy *et al.*, 2012; Props *et al.*, 2018) and at the metabolic level through MALDI‐TOF MS (Sala‐Comorera *et al.*, 2016; Sandrin and Demirev, 2018). A key aspect in this approach concerns knowledge of the contribution of deterministic processes, which can be monitored and controlled, to the microbial community dynamics. Subsequently, the variance in community dynamics not explained by deterministic processes can be attributed to influence of stochastic processes.

Benchmarking of microbial community dynamics is essential to distinguish between a system with acceptable background dynamics, as observed in anaerobic digestion (Klang *et al.*, 2015) and activated sludge (Mielczarek *et al.*, 2012; Mielczarek *et al.*, 2013; Ju and Zhang, 2015) systems, and uncontrollable domination of stochasticity over deterministic effects. Conventional microbial community dynamics at steady state ranges between 20 and 30% change in microbial community composition in a 14‐day window in anaerobic digestion (Pycke *et al.*, 2011; De Vrieze *et al.*, 2016) and activated sludge (Wang *et al.*, 2010) systems. However, the background degree of change depends on multiple factors and should be evaluated for each case specifically. For example, the dynamics of the ammonia oxidizing bacterial community was twice as high in a membrane bioreactor, compared to a sequential batch reactor, both showing functional stability and operating under similar conditions (Wittebolle *et al.*, 2008).

Drinking water supply systems, as an example of a process related to direct human usage, require a more direct monitoring of stochasticity beyond the DNA level to phenotypic and/or activity changes for which flow cytometry (De Roy *et al.*, 2012) and MALDI‐TOF MS (Sala‐Comorera *et al.*, 2016) could be suitable methods. Such flow cytometry methods can even be extended to more complex systems, such as anaerobic digestion (Dhoble *et al.*, 2016) and activated sludge (Günther *et al.*, 2012), indicating the progress in accurate fingerprinting techniques. Overall, the key aspect of these fingerprinting techniques is their moderate level of cost and rapid nature to gain a real‐time view on the status of the stochasticity impact.

Key to the validity of the fingerprinting approach is knowledge on (i) the initial status of the microbial community, (ii) the deterministic processes (potentially) influencing the microbial community and (iii) the (change in) input streams, e.g., the absence of pathogens and ARGs, for which the ‘omics’ or targeted methods are needed, as discussed above. Taking these three conditions into consideration, if fingerprinting methods do not indicate a deviation from the background variation, no additional analyses nor direct actions are needed. Deviations from the background dynamics profile warrant further investigation to pinpoint potential stochastic hazards and take necessary actions.

Even though these fingerprinting techniques provide an important overall view on the microbial community dynamics and distinguish background dynamics from strong deviations or sudden changes, they fail to detect the gradual potential influx of pathogens. Hence, identification at the lowest most precisely possible taxonomic level is mandatory to identify potential pathogens. For this, 16S rRNA gene amplicon sequencing falls short on two levels, i.e., (i) the limited depth of phylogenetic identification (Poretsky *et al.*, 2014) and (ii) the inability to accurately identify rare (pathogenic) species (Huse *et al.*, 2010; Kunin *et al.*, 2010), in contrast to metagenome analyses. Metagenome analysis has the potential to identify genes that are possibly related to antibiotic resistance or to the production of toxins, e.g., in reclaimed water distribution systems (Garner *et al.*, 2018), with co‐occurrences across ecosystems (Li *et al.*, 2015). However, their presence in the metagenome does not necessarily imply their actual expression.

Monitoring of expression or translation of undesirable genes, e.g., related to toxicity, can take place at the RNA, protein and general metabolic level, either focussed on the general microbial community level or to specific targets. To gain an overall view on the microbial community, ‘omics’ approaches are essential to map the actual ‘performance’ of the microbial community to stochasticity. A metatranscriptomic approach has been shown useful to identify the transcription of ARGs in activated sludge systems (Liu *et al.*, 2019) and to characterize host–microbe pathogenic interactions (Hampton‐Marcell *et al.*, 2013). Yet, this still presents multiple methodological challenges (Asante and Osei Sekyere, 2019). Similarly, metaproteomic and metabolomic analyses could assist in the characterization of stochastic hurdles in microbiomes. Based on this information, specific targets can be identified for which suitable methods can be optimized, for example using real‐time PCR methods, for detecting ARGs and/or pathogens in wastewater treatment systems (Volkmann *et al.*, 2004; Tao *et al.*, 2014), manure treatment systems (Yu *et al.*, 2005) and artificial groundwater recharge systems (Böckelmann *et al.*, 2009), as well as their expression and/or activity.

Overall, monitoring of stochasticity, especially related to the key impact of invasion, can be considered challenging in microbiomes, in contrast to single culture‐based systems. A combination of technologies is essential to monitor stochasticity in microbiomes. Fingerprinting at different levels as such can be considered insufficient, yet can serve as the first stochastic ‘smoking gun’ to push for further elucidation, for which the ‘omics’ can provide subsequent suitable information. This information then needs to be translated into accurate methods that can pinpoint stochasticity‐related microbial flaws in the system. An integrated case‐by‐case approach should be considered, as discussed in Setting the boundaries on stochasticity: tolerance versus assurance, to monitor stochasticity for which the application range needs to be investigated.

### Setting the boundaries on stochasticity: tolerance versus assurance

**Fig. 3:**
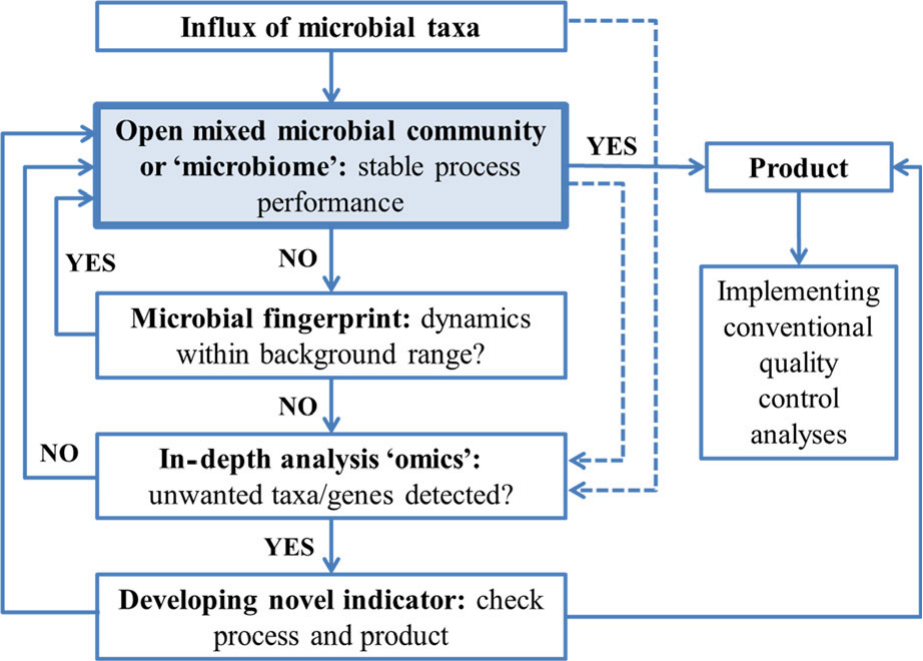
Schematic overview of the different steps for stochasticity monitoring and control in microbiomes. Full lines represent actual decisions and actions, while dashed lines represent flows of information.

Technological developments in the field of high‐throughput sequencing in the last decades have provided us with the tools for detailed monitoring of stochasticity in a microbiome, as discussed above. However, the time‐consuming and complex nature, as well as associated costs of high‐throughput techniques, are bottlenecks for full‐scale application and warrant consideration for the frequency and depth of application. Nonetheless, high‐throughput sequencing methods are becoming cheaper, faster and more directly accessible, e.g., with the development of the MinION™ device (Quick *et al.*, 2014) and gradually find their way into different fields, such as clinical microbiology (Deurenberg *et al.*, 2017). Here, we propose an integrated approach (Fig. 3) to monitor stochasticity to keep the potential risks towards human health and process disturbance as low as reasonably achievable (ALARA).

Initial screening of the degree of stochasticity in function of time (e.g. dynamics) can take place through basic fingerprinting methods, as mentioned earlier. The selection of the appropriate fingerprinting method depends on the system, as amplicon sequencing at the DNA level can be considered suitable for processes in which the outcome does not directly link to human consumption, such as anaerobic digestion and activated sludge systems.

At present, such an approach that relies on microbial fingerprinting represents a balance between the need for costly and time‐consuming analyses and the reliability of timely screening, even though this does not provide 100% assurance of process/product safety. The industrial practice of using microbiomes generally allows that the ‘contaminants’ can be present at a factor 10 000 lower than the functional species of interest. A clear example of how the complete absence of unwanted species is an unfeasible imposition for full‐scale industrial processes using microbiomes is provided by the production of microbial protein from natural gas (Hamer, 2010). In this case, the final product (BioProtein^®^) obtained by growing a single culture of *Methylococcus capsulatus* under semi‐sterile conditions was eventually characterized by the presence of heterotrophic bacterial strains, which fill a key functional niche necessary to maintain acceptable process performances, i.e., the degradation of the organic carbon produced by *Methylococcus capsulatus* (Bothe *et al.*, 2002). Such cooperation and the possibility that newly appearing species might change over time did not hinder the regulatory approval of BioProtein^®^ as a feed additive. The same product is now being developed for human food purposes (Strong *et al.*, 2015). Other less obvious examples are reflected in Belgian Gueuze beers (Spitaels *et al.*, 2014), traditional Icelandic fermented fish, i.e., hákarl (Osimani *et al.*, 2019), traditional cheeses (Montel *et al.*, 2014) and salami (Połka *et al.*, 2015), none of which contain a defined microbiome, but also contain few or no pathogenic interference in the processing stage or final product.

Overall, engineered processes involving microbiomes are essential for waste(water) treatment, energy recovery, drinking water supply and food processing, and, therefore, cannot be eliminated from our present society. Complete elimination of all risks in microbiomes is impossible (tolerance), due to the stochastic nature of the microbiome. However, if the necessary precautions and above‐mentioned level of monitoring, e.g., on site via the MinION™ device (Maestri *et al.*, 2019), are considered (assurance), microbial processes will continue to play a crucial role in our efforts to pursue the Sustainable Development Goals for a more sustainable future.

## Conflict of interest

None declared.
